# The Value of C-reactive Protein/Albumin Ratio in the Prediction of Contrast-Induced Nephropathy in Emergency Department Patients

**DOI:** 10.7759/cureus.39230

**Published:** 2023-05-19

**Authors:** Tuğçe Zengin Temel, Dilay Satilmis, Burcu G Yavuz, Mustafa Ahmet Afacan, Sahin Colak

**Affiliations:** 1 Department of Emergency Medicine, Beykoz State Hospital, Istanbul, TUR; 2 Department of Emergency Medicine, University of Health Sciences Sultan 2. Abdulhamid Han Training and Research Hospital, Istanbul, TUR; 3 Department of Emergency Medicine, University of Health Sciences Haydarpasa Numune Training and Research Hospital, Istanbul, TUR

**Keywords:** car, contrast media, inflammation, c-reactive protein to albumin ratio, contrast-induced nephropathy

## Abstract

Background: Contrast-induced nephropathy (CIN) is the third most common cause of acute renal failure in hospitalized patients and is an important cause of prolonged hospital stay, morbidity, and mortality. We aimed to investigate the effectiveness and sufficiency of the prognostic capacity of the inflammatory biomarkers C-reactive Protein (CRP) and albumin ratio (CAR) in predicting the development of CIN in patients undergoing contrast-enhanced computed tomography (CT) imaging in the emergency department (ED).

Methods: This study was performed on patients whose laboratory values ​​could be reached within 48 hours after contrast-enhanced CT imaging in the emergency department of our hospital. The patients were divided into two groups as those with and without CIN according to their increased creatinine levels. Its effectiveness in detecting the development of CIN in the early period was evaluated comparatively.

Results: One hundred and twenty-five patients were included. CIN developed in 10.4% of the patients. The CAR was 0.19 (IQR: 0.17-0.33) in the group with CIN and 0.02 (IQR: 0.01-0.06) in the group without CIN; and the difference between the two groups was significant (p<0.001). In multivariate logistic regression analysis, it was found that the CAR increased as an independent risk factor for CIN (OR: 2.326; 95% CI: 1.39-3.893; p=0.001).

Conclusions: We think that early identification of patients who may develop CIN through the CAR in EDs and early initiation of treatment for CIN may affect the morbidity-mortality rate and reduce the duration of hospitalization and treatment costs.

## Introduction

Contrast-induced nephropathy (CIN) is defined as an elevation of more than 0.5 mg/dL in serum creatinine level 48 hours after administration of intravascular contrast media (CM) or an elevation of 25% or more compared to baseline serum creatinine level [1,2]. In CIN, serum creatinine level rises in the 24th-48th hours after CM exposure, peaks on the third-fifth day, and decreases to the normal level within one to three weeks. The third most common cause of acute kidney injury in hospitalized patients is CIN [3]. Approximately 150,000 patients are diagnosed with CIN every year in the world, and 1% of them need dialysis and have prolonged hospitalization [4].

In addition to the renal medullary hypoxia and direct toxic effects of CM on renal tubular cells, which are the pathophysiological mechanisms in the development of CIN, inflammation is also shown among the causes of CIN occurrence [2,5,6]. In studies, C-reactive protein (CRP), neutrophil, leukocyte count, neutrophil to lymphocyte ratio (NLR), and procalcitonin levels were associated with the development of CIN as a positive marker of inflammation, whereas albumin level was associated as a negative marker [7,8]. However, to our knowledge, there is no study on the effectiveness of the CRP/albumin ratio (CAR) in predicting the development of CIN in the early period in patients who underwent contrast-enhanced computed tomography (CT) imaging in the emergency department (ED). Therefore, in this study, our primary endpoint was to investigate the effectiveness and sufficiency of the early prognostic capacity of CAR, which is an acute-phase reactant and is known to be associated with poor outcomes in predicting the development of CIN, and our secondary endpoint is to evaluate mortality rates in patients.

## Materials and methods

Our study was a single-center, retrospective, observational trial. A total of 125 patients, who applied to the Haydarpasa Numune Training and Research Hospital Emergency Medicine Clinic between January 1, 2018 and January 1, 2021, whose first examination was performed in the ED, whose tests and treatment was planned, and who were scheduled for contrast-enhanced CT angiography imaging, whose laboratory values ​​​​were able to reach at 48 hours, were included in this study. Patients with active malignancy, chronic liver disease, autoimmune disease, end-stage renal disease (glomerular filtration rate (GFR) <15 or dialysis patient), and chronic rheumatological disease that may cause systemic and local infections and affect the results, and patients who were referred to an external center were excluded from this study.

The clinical manifestations, physical examination findings, and blood samples of the patients during ED admission were noted down. All patients who received 100-150 mL of non-ionic, low-osmolality intravenous (IV) CM (Iohexol-Omnipaque) and whose serum creatinine concentrations were measured before and 48 hours after CM administration were included in this study. Demographic and clinical data were analyzed to identify predictors of CIN development. GFR levels were calculated using the Modification of Diet in Renal Disease Study (MDRD) formula (186 x (SCr) - 1.154 x (age) - 0.203 x (0.742 if female) x (1.212 if black)) [9].

Serum creatinine, CRP, and albumin levels were recorded. Cut-off values for CRP and albumin were 0 to 5 mg/L and 35 to 55 mg/dL, respectively. The CAR was calculated by dividing the serum CRP level by the serum albumin level. Analyses of blood samples 48 hours after CT angiography were used to evaluate CIN. An elevation of more than 0.5 mg/dL in the serum creatinine level or an elevation of 25% or more compared to the baseline serum creatinine level was considered significant for CIN.

Ethical consideration

Ethical approval was obtained from the ethics committee of the Haydarpasa Numune Training and Research Hospital’s Ethics Committee 2021/171 (HNEAH-KAEK 2021/KK/171). It was conducted in compliance with the principles of the Declaration of Helsinki. The hospital ethics committee waived written informed consent because this study was retrospective and evaluated only the clinical data of the patients and did not involve any potential risk. The results of this study are reported according to the Strengthening the Reporting of Observational Studies in Epidemiology (STROBE) recommendations [10].

Statistical analysis

Categorical variables in this study were presented as frequency (n) and percentage (%) and analyzed via Pearson's chi-square test and Fisher's exact test. Normally distributed continuous variables were expressed as mean ± standard deviation (SD) and non-distributed ones as median (IQR: 25th-75th percentile). The Shapiro-Wilk test was used to check whether the variables fit the normal distribution. Mann-Whitney U test and the independent t-test were used to determine the difference between continuous variables depending on the presence of CIN. Receiver operating characteristic (ROC) analysis was conducted to differentiate patients according to CRP, lymphocyte, CAR, and NLR values and determine the cut-off point in identifying the development of CIN. Analysis results were presented with AUC, cut-off points, sensitivity, specificity, and 95% confidence intervals. Optimal cut-off points of the variables were determined by the Youden index. Multivariate logistic regression analysis was conducted to identify risk factors independently associated with CIN. The obtained results were expressed with odds ratio (OR) and 95% confidence intervals. Data analysis was conducted using the IBM SPSS 23.0 software (IBM Corp., Armonk, NY). The results were considered significant at p<0.05.

## Results

In the study, 125 patients with a mean age of 65.96±14.44 years and 74 (59.2%) men were included. 52.8% of the patients had hypertension (HT), 32% had diabetes mellitus (DM), 26.4% had coronary artery disease (CAD), and 24.8% had cerebrovascular disease (CVD). Of the patients included in the study, 13 (10.4%) developed CIN. The mortality rate was significantly higher in patients with CIN (p=0.004). Eight (6.4%) of 125 patients died. The mortality rate of with CIN group was 30.8% and without CIN group was 3.6%. The results regarding the demographic and clinical characteristics of the patients are presented in Table 1.

**Table 1:** Demographic and clinical features Abbreviations: CIN: Contrast-induced nephropathy, ED: Emergency department

Variables	All patients	With CIN	Without CIN	P-value
Number of patients, (%)	125	112(89.6)	13(10.4)	-
Age (years)	65.96±14.44	65.74±14.64	67.85±13.04	0.621
Sex				
Male	74(59.2)	66(58.9)	8(61.5)	0.856
Female	51(40.8)	46(41.1)	5(38.5)	
Coexisting diseases				
Diabetes mellitus	40(32)	36(32.1)	4(30.8)	0.999
Hypertension	66(52.8)	57(50.9)	9(69.2)	0.210
Coronary artery disease	33(26.4)	29(25.9)	4(30.8)	0.743
Cerebrovascular disease	31(24.8)	24(21.4)	7(53.8)	0.017
Complaints				
Syncope	12(9.6)	10(8.9)	2(15.4)	0.362
Dysarthria	37(29.6)	35(31.3)	2(15.4)	0.342
Dizziness	31(24.8)	28(25)	3(23.1)	0.999
Headache	4(3.2)	3(2.7)	1(7.7)	0.359
Facial asymmetry	9(7.2)	8(7.1)	1(7.7)	0.999
Vomiting	9(7.2)	7(6.3)	2(15.4)	0.236
Defect of vision	10(8)	8(7.1)	2(15.4)	0.278
Change of consciousness	7(5.6)	6(5.4)	1(7.7)	0.546
ED final status				
Discharged	12(9.6)	12(10.7)	0(0)	0.613
Hospitalization	113(90.4)	100(89.3)	13(100)	
Mortality	8(6.4)	4(3.6)	4(30.8)	0.004

The CRP values of patients who developed CIN (7.5 (IQR: 7-12.9)) were significantly higher than patients who did not develop CIN (1 (IQR: 0.4-2.35)) (p<0.001). The median CAR was 0.19 (IQR: 0.17-0.33) in the group with CIN and 0.02 (IQR: 0.01-0.06) in the group without CIN, the difference between the two groups was significant (p<0.001).

The median lymphocyte count was 1.25 (IQR: 0.92-2.03) in patients who developed CIN, while it was calculated to be 1.96 (IQR: 1.39-2.68) in patients who did not develop CIN. Lymphocyte values were lower in patients who developed CIN (p=0.020). Neutrophil-lymphocyte ratio (NLR) values were significantly higher in patients with CIN than in patients without CIN (5.34 [IQR: 3.14-8.49] and 2.67 [IQR: 1.97-4.32]; p=0.028) (Table 2).

**Table 2:** Laboratory findings of the patients Findings were given with the median (IQR). Mann-Whitney U test. Abbreviations: CIN: Contrast-induced nephropathy, GFR: Glomerular filtration rate, CRP: C-reactive protein, BUN: Blood urea nitrogen, WBC: White blood cell, HGB:Hemoglobin, PLT:platelet, CAR: C-reactive protein/albumin ratio, NLR: Neutrophil-lymphocyte ratio

Variables	All patients (n=125)	With CIN (n=112)	Without CIN (n=13)	p-value
GFR	81.59(71.84-98.84)	83.63(72.8-100.11)	80.89(63.32-92.01)	0.217
CRP	1.15(0.5-5.28)	1(0.4-2.35)	7.5(7-12.9)	<0.001
Albumin	40.8(37-43)	41(37-43.67)	39(38-41.47)	0.425
BUN	16(13.13-21)	15.97(13.08-20.42)	18.03(17.3-24.25)	0.096
Creatinine	0.85(0.73-1.03)	0.84(0.73-1)	1.1(0.83-1.18)	0.053
WBC	8.3(7.05-9.86)	8.3(6.98-9.73)	8.69(7.54-11.19)	0.467
Neutrophil	5.6(4.43-6.94)	5.5(4.43-6.9)	6.36(6.25-7.46)	0.086
Lymphocyte	1.93(1.28-2.53)	1.96(1.39-2.68)	1.25(0.92-2.03)	0.020
HGB	12.9(12-14.8)	13.35(12.2-14.8)	12.2(11-13.4)	0.159
PLT	234(184.5-289.5)	224(183-288)	253(238-295)	0.140
CAR	0.03(0.01-0.14)	0.02(0.01-0.06)	0.19(0.17-0.33)	<0.001
NLR	2.72(2.02-5.07)	2.67(1.97-4.32)	5.34(3.14-8.49)	0.028

Multivariate logistic regression analysis was conducted to identify the risk factors that independently affect the development of CIN in patients, and the results are presented in Table 2. Two separate models were established since there is a strong correlation between CRP and CAR. In Model 1, CRP was independently associated with CIN. In Model 2, CAR was identified as an independent risk factor for CIN occurrence. It was determined that high CAR increased the risk of developing CIN (OR: 2.326; 95% CI: 1.39-3.893; p=0.001).

**Figure 1:**
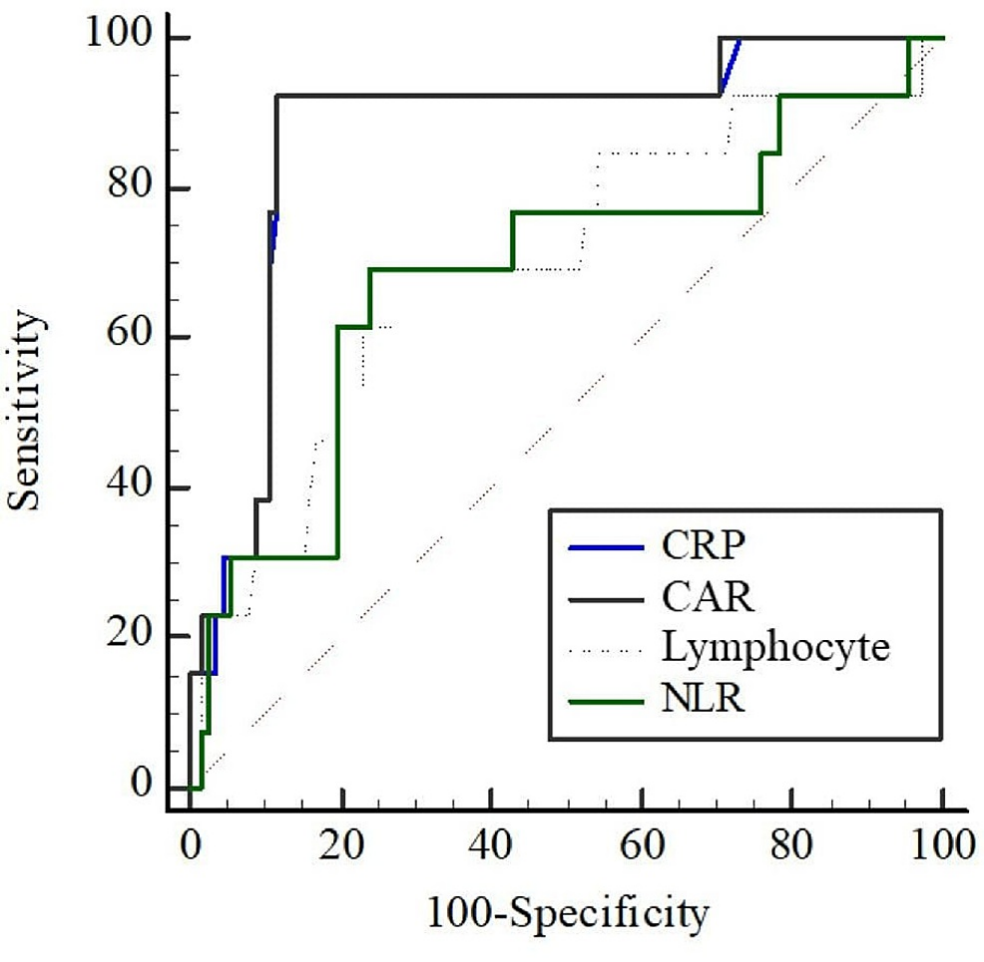
Receiver operating characteristic (ROC) curves Abbreviations: CRP: C-reactive protein, CAR: C-reactive protein/albumin ratio, NLR: Neutrophil-lymphocyte ratio

When we compared the differential performance of the CAR for CIN development with other biomarkers, it was significantly higher than lymphocyte (AUC=0.874 and AUC=0.697; p=0.025) and NLR (AUC=0.874 and AUC=0.686; p=0.023) and was statistically similar to CRP (AUC=0.872 and AUC=0.874; p=0.545) (Figure 1).

## Discussion

Inflammation has a key role in predicting CIN [7]. Positive acute-phase reactant CRP and negative acute-phase reactant albumin have been defined as good indicators of inflammation in the predictive effectiveness of inflammatory biomarkers for CIN [11,12]. In the current study, unlike other studies, we investigated the effectiveness of inflammatory biomarkers in predicting CIN in patient groups administered IV CM in ED. In this respect, we scrutinized the relationship of the ratio of both biomarkers to each other with the development of CIN compared to evaluations of CRP and albumin alone.

Studies have demonstrated that CIN occurs at a higher rate in male patients than female patients, but no correlation was found between gender and the occurrence of CIN [12-14]. Likewise, in our study, 61.5% of the CIN patients were male and 38.5% were female, and the rate of male patients was higher than that of females. In addition, consistent with the literature, no significant correlation was found between gender and the occurrence of CIN (p=0.856).

In studies on CIN and comorbidities, HT, DM, and hyperlipidemia ranked among the top three comorbid diseases [11,15]. Consistent with previous studies, HT was the most common comorbid disease with 69.2% in our study, but unlike other studies, CVD (53.8%) ranked second, while CAD and DM ranked third (30.8%).

In a study by Murat et al., in which 890 patients were included, the albumin level and the development of CIN were evaluated in patients who underwent percutaneous coronary intervention (PCI), and it was revealed that the serum albumin level was lower in patients who developed CIN than in those who did not and that low serum albumin levels could predict the development of CIN [8].

In the study by Yaşar et al. on the development of CIN in acute ischemic stroke patients receiving percutaneous treatment, CIN developed in 17% of the patients. The CAR was significantly associated with CIN predictivity with a sensitivity of 80.7 % and a specificity of 92.6% [15]. Consistent with previous studies, in our study, the predictive value of the CAR in predicting CIN was significantly higher, with a sensitivity of 92.31% and a specificity of 88.39%. We consider that this situation supports that the predictive values of isolated laboratory results may be affected by comorbid conditions in patients and that the use of more than one laboratory value to strengthen the confidence interval increases the diagnostic value.

In the study by Evola et al. on risk factors for CIN, the findings showed that 17.7% of the patients developed CIN, and the pre-procedural CRP level was significantly higher in the group that developed CIN compared to the group that did not develop CIN (p=0.03) [14]. Likewise, in the study conducted by Satilmis et al. on the effectiveness of biomarkers in predicting the development of CIN in non-STEMI patients who underwent PCI, it was demonstrated that 9.2% of the patients developed CIN, and when ROC analyses were evaluated, the CAR, one of the biomarkers, was significant (AUC: 0.802). In our study, consistent with other studies, the CAR was significant in predicting the development of CIN, according to ROC analysis (AUC: 0.874) [12]. In this study, we also evaluated the effectiveness of inflammatory biomarkers lymphocyte and NLR in predicting CIN and compared them with CAR. Similarly, in our study, the effectiveness of a decrease in lymphocyte level and an increase in NLR in predicting CIN was significant (p=0.020 and p=0.028, respectively) [16-19]. When we compared the differential performance of the CAR with other biomarkers, we found that it was significantly higher than lymphocyte (AUC=0.874 and AUC=0.697; p=0.025) and NLR (AUC=0.874 and AUC=0.686; p=0.023).

In the study of Narula et al., in which they evaluated the mortality rate in 479 patients who developed CIN, the 30-day mortality rate of the patients was 8%, and the rate of development of mortality was significantly higher in patients with CIN [13]. In our study, the mortality rate (30.8%) was higher in patients with CIN than in patients without CIN, and a significant difference was found between the two groups (p=0.004). We think that the high mortality rate in patients with CIN in our study is due to the higher rate of critical patient follow-up in EDs and the use of contrast-enhanced imaging in the differential diagnosis of critically ill patients.

In our study, the results obtained on the effectiveness of biomarkers in predicting CIN in the early period were higher and more significant than in other studies in the literature. We consider that this situation could increase the value of using easily accessible biomarkers in patient follow-up and discharge decisions. Moreover, there is no adequate comparison and evaluation study in the literature to predict early CIN in ED. Thus, our study should be regarded as a biomarker study that could be used to identify CAR in the early period in ED clinics with high patient density.

Study limitations

The present study has several limitations. First of all, it is an important limitation that this study is single-centered and retrospective. Secondly, since the number of hospitalization clinics in our hospital was insufficient, patients who were given contrast were referred to external centers. There is a lack of data in the included patient sample. However, all consecutive patients meeting the criteria in the ED were included, thereby limiting patient selection bias. To our knowledge, the correlation between CAR and CIN in the ED patient group has not been reported before. Therefore, further studies with larger samples and randomized patients are needed.

## Conclusions

In our study, it has been revealed that CRP/CAR, which are biomarkers that respond inversely to each other regarding inflammation, increased the diagnostic value for CIN compared to the evaluation of albumin and CRP, separately. The CAR was significantly higher in CIN patients. We think that early identification of CIN will contribute significantly to the length of hospital stay, mortality, and morbidity in the ED.

## References

[1] Cronin RE (2010). Contrast-induced nephropathy: pathogenesis and prevention. Pediatr Nephrol.

[2] Porter GA (1989). Contrast-associated nephropathy. Am J Cardiol.

[3] Hou SH, Bushinsky DA, Wish JB, Cohen JJ, Harrington JT (1983). Hospital-acquired renal insufficiency: a prospective study. Am J Med.

[4] Feldkamp T, Kribben A (2008). Contrast media induced nephropathy: definition, incidence, outcome, pathophysiology, risk factors and prevention. Minerva Med.

[5] Heyman SN, Reichman J, Brezis M (1999). Pathophysiology of radiocontrast nephropathy: a role for medullary hypoxia. Invest Radiol.

[6] Kwasa EA, Vinayak S, Armstrong R (2014). The role of inflammation in contrast-induced nephropathy. Br J Radiol.

[7] Kan WC, Huang YT, Wu VC, Shiao CC (2021). Predictive ability of procalcitonin for acute kidney injury: a narrative review focusing on the interference of infection. Int J Mol Sci.

[8] Murat SN, Kurtul A, Yarlioglues M (2015). Impact of serum albumin levels on contrast-induced acute kidney injury in patients with acute coronary syndromes treated with percutaneous coronary intervention. Angiology.

[9] Hojs R, Bevc S, Ekart R, Gorenjak M, Puklavec L (2011). Kidney function estimating equations in patients with chronic kidney disease. Int J Clin Pract.

[10] Karabağ Y, Çağdaş M, Rencuzogullari I (2019). The C-reactive protein to albumin ratio predicts acute kidney injury in patients with ST-segment elevation myocardial infarction undergoing primary percutaneous coronary intervention. Heart Lung Circ.

[11] von Elm E, Altman DG, Egger M, Pocock SJ, Gøtzsche PC, Vandenbroucke JP (2008). The strengthening the Reporting of OBservational studies in Epidemiology (STROBE) statement: Guidelines for reporting observational studies. J Clin Epidemiol.

[12] Satilmis S, Karabulut A (2020). Value of C-reactive protein/albumin ratio in predicting the development of contrast-induced nephropathy in patients with non-ST elevation myocardial infarction. Angiology.

[13] Narula A, Mehran R, Weisz G (2014). Contrast-induced acute kidney injury after primary percutaneous coronary intervention: results from the HORIZONS-AMI substudy. Eur Heart J.

[14] Evola S, Lunetta M, Macaione F (2012). Risk factors for contrast induced nephropathy: a study among Italian patients. Indian Heart J.

[15] Yaşar Yaşar, E E (2022). Predictive value of C-reactive protein/Albumi̇n ratio in the development of contrast-induced nephropathy in patients with acute ischemic stroke treated percutaneously. İnönü Üniversitesi Sağlık Hizmetleri Meslek Yüksek Okulu Dergisi.

[16] Zehir R, Sarak T, Zehir S (2017). Platelet-to-lymphocyte ratio predicts contrast-induced nephropathy in acute myocardial infarction. Turk Clin Lab.

[17] Butt K, D'Souza J, Yuan C, Jayakumaran J, Nguyen M, Butt HI, Abusaada K (2020). Correlation of the neutrophil-to-lymphocyte ratio (NLR) and platelet-to-lymphocyte ratio (PLR) with contrast-induced nephropathy in patients with acute coronary syndrome undergoing percutaneous coronary interventions. Cureus.

[18] Kaya A, Kaya Y, Topçu S (2014). Neutrophil-to-lymphocyte ratio predicts contrast-induced nephropathy in patients undergoing primary percutaneous coronary intervention. Angiology.

[19] Yuan Y, Qiu H, Hu X (2017). Predictive value of inflammatory factors on contrast‐induced acute kidney injury in patients who underwent an emergency percutaneous coronary intervention. Clin Cardiol.

